# Genetics of Keratoconus: Where Do We Stand?

**DOI:** 10.1155/2014/641708

**Published:** 2014-08-28

**Authors:** Khaled K. Abu-Amero, Abdulrahman M. Al-Muammar, Altaf A. Kondkar

**Affiliations:** ^1^Ophthalmic Genetics Laboratory, Department of Ophthalmology, College of Medicine, King Saud University, P.O. Box 245, Riyadh 11411, Saudi Arabia; ^2^Glaucoma Research Chair, Department of Ophthalmology, College of Medicine, King Saud University, P.O. Box 245, Riyadh 11411, Saudi Arabia; ^3^Department of Ophthalmology, College of Medicine, University of Florida, Jacksonville, FL 32209, USA

## Abstract

Keratoconus is a progressive thinning and anterior protrusion of the cornea that results in steepening and distortion of the cornea, altered refractive powers, and reduced vision. Keratoconus has a complex multifactorial etiology, with environmental, behavioral, and multiple genetic components contributing to the disease pathophysiology. Using genome-wide and candidate gene approaches several genomic loci and genes have been identified that highlight the complex molecular etiology of this disease. The review focuses on current knowledge of these genetic risk factors associated with keratoconus.

## 1. Introduction

Keratoconus is a corneal ectatic disease that results in bilateral and asymmetrical corneal distortion, altered refractive powers, and reduced vision. The disease usually manifests itself during the late teens or early twenties and shows a slow progression for the next decade or two. The clinical signs of keratoconus are highly variable depending on the stage of progression of the disease and may include stromal thinning, conical protrusion, Fleischer's ring, Vogt's striae, increased visibility of nerve fibres, and rupture in Bowman's layer [1–3].

Keratoconus is known to affect all ethnicities but its incidence exhibits geographical variability plausibly due to forme fruste or subclinical forms of the disease, differences in diagnostic methods and criteria, or differences in genetic variations in such populations [4]. Different population-based studies have evaluated the prevalence of keratoconus among different ethnic groups and the rates are found to be variable. The estimated incidence of keratoconus varies between 1 in 500 and 1 in 2,000 individuals in the general population and the estimated prevalence is reported to be 54.5 per 100,000 [1, 5]. Studies suggest that the prevalence and incidence rates are much higher among Asians than the Caucasian population [6, 7]. In a study undertaken in the Midlands, UK, a prevalence of 4 : 1 and an incidence of 4.4 : 1 were reported in Asians compared to the white Caucasians [6] while another UK study conducted in Yorkshire found that the incidence was 7.5 times higher in Asians as compared to the whites [7]. Population studies in the Middle East (including the Arabs and non-Arabs) suggest that the incidence of keratoconus is between 20/100,000 and 24.9/100,000 [8, 9] which is comparable to that observed in the Asian-Pacific population [6, 7]. However, in one study from India, the prevalence was reported to be as high as 2.3% (2,300 per 100,000) [10]. The mean age of onset of keratoconus is reported to be 39.3 years [11]. The severity of the disease also varies with race and was reported to be 4.4 and 7.5 times greater among Asian group compared to white Caucasians [6, 7]. And although exact figures are not available, keratoconus is believed to be overrepresented and more aggressive in the Maori and Polynesian populations of New Zealand [12].

Keratoconus is a multifactorial disease involving complex interaction of both genetic and environmental factors that contribute to the disease manifestation. As with any other diseases of complex etiology, differentiation between association, cause, and effect is very challenging and varies between individuals. Although keratoconus affects both genders, most of the studies suggest a preponderance of men over women [13–15]. In the study by Georgiou et al. keratoconus was 2.6 times more common in males than in females [7] and five times more common in men than women by Millodot et al. [14]. However, there are studies that either reported no differences among genders [16] or showed a greater prevalence in females [17]. In some individuals keratoconus may be entirely associated with currently well recognized environmental influences such as contact lens wear, eye rubbing, atopy of the eye, and those related to increased oxidative damage, such as ultraviolet light and yet in another may be solely controlled by genetic mechanisms exhibiting Mendelian inheritance pattern [1, 18]. Studies supporting the role of consanguinity as a risk factor for keratoconus have also been documented [7, 19] providing strong evidence of genetic contribution to the disease and may be relevant in communities practicing consanguineous marriages, such as in Saudi Arabia. However, a majority of keratoconus cases occur as a result of the genetic predisposition triggered by environmental factors [20]. This review summarizes the current knowledge on genetic aspects of keratoconus.

## 2. Role of Heredity in Keratoconus

A majority of keratoconus cases are sporadic; however, autosomal dominant with reduced penetrance and autosomal recessive mode of inheritance have also been documented [21–24]. First-degree relatives are at much higher risk of the disease than the general population [5, 25]. Monozygotic twins show a high concordance of keratoconus with a greater similarity of phenotypes indicating a strong role of genetic component(s) in the disease phenotype [26]. These data provide strong evidence to support the role of heredity in keratoconus.

## 3. Genetic Studies in Keratoconus

Corneal topographical assessment,* in vivo* confocal microscopy, Placido disk analysis, and slit lamp biomicroscopy have greatly aided the diagnosis of keratoconus [27] but are often highly variable and difficult to interpret in young individuals with mild symptoms. Identification of specific genetic markers may thus be a valuable tool in clinical diagnostics. Multiple genomic approaches have been used to identify chromosomal loci and genes involved in keratoconus.

## 4. Linkage Studies

Linkage analysis is a powerful tool to map susceptible genetic loci and has been utilized at genome-wide level in keratoconus. To date, 17 distinct genomic loci have been mapped for keratoconus indicating that there exists high degree of genetic heterogeneity in this disease [28] (Table 1). Unfortunately, only three of these loci, namely, 5q21, 5q32, and 14q11, have been replicated independently [24]. However, using this approach in large extended pedigrees has led to the identification of two potential genes (*MIR184* and* DOCK9*) associated with keratoconus as described below (Table 2).

Using linkage analysis [21] and fine mapping [29] a 5.5 Mb region of chromosome 15q22-q24a was identified in a single extended pedigree from Northern Ireland with hereditary keratoconus related with anterior capsular cataract. With deep sequencing using next-generation sequencing technology, three novel variants were identified recently in* DNAJA4*,* IREB2*, and* MIR184* genes that segregated with the phenotype in this family. A mutation (r.57 c>u) in the micro-RNA (miRNA) gene* MIR184* (OMIM 613146) was considered to be the most likely cause [30]. miRNA regulate more than 60% of known genes by the mechanism of mRNA degradation and inhibition of protein translation by binding to the 3′ UTR region of the gene and are known to affect disease phenotypes [31].* MIR184* is abundantly expressed in the cornea and lens epithelia and mutation in the seed region of* MIR184* is likely to affect its function. Consistent with this finding, subsequent studies identified the same mutation in families with EDICT syndrome (endothelial dystrophy, iris hypoplasia, congenital cataract, and stromal thinning) [32] and dominant congenital cataract associated with corneal phenotype [33]. However, a study in 780 keratoconus patients could identify* MIR184* variants in only 0.25% (two) patients indicating that variants in* MIR184* gene may not account for isolated keratoconus [34].

In another study in large keratoconus family of Ecuadorian origin Gajecka and colleagues identified a region of 5.59 Mb on chromosome 13q32 consisting of 25 genes using whole-genome single nucleotide polymorphism (SNP) 250 K array [35]. Sequencing of eight candidate genes led to the identification of a novel variant c.2262a>c (Gln745His) in the* dedicator of cytokinesis 9* (*DOCK9*; OMIM 607325) gene that segregated in the family and was absent in ethnically matched controls [36].* DOCK9* is expressed in the cornea and specifically activates CDC42, a G-protein. Although the exact mechanism by which this mutation may cause keratoconus is not clear, it is known that the mutation is located in DHR1 domain which binds phospholipids and may affect recruitment of the protein to the membrane and it is predicted to be “possibly damaging” [36]. Furthermore, this locus contains additional genes,* IPO5* (*importin 5*; OMIM 602008) and* STK24* (*serine/threonine kinase 24*; OMIM 604984), the exact role of which in keratoconus pathogenesis is not known yet. A recent study in Polish patients with sporadic keratoconus, however, reported that keratoconus-related sequence variants in these genes (*DOCK9, IPO5, *and* STK24*) were present only in small number of studied patients indicating that these genes may have minor roles to play [37].

Several other loci have been reported in keratoconus families among different population (listed in Table 1) [18, 23, 24, 38–43]. A locus on 17p13 was reported in a two-generation Pakistani family with autosomal recessive Leber congenital amaurosis and keratoconus [44]. Li et al. reported several regions of linkage on chromosomes 4, 5, 9, 12, and 14 using two-stage genome-wide linkage scan in keratoconus sib pair families of white and Hispanic origin [41]. Similarly, a genome-wide linkage scan in a large Australian pedigree identified two regions of linkage at chromosomal regions 1p36.23-36.21 and 8q13.1-q21.11 [18]. Moreover, Bisceglia et al. provided evidence of linkage replication in keratoconus at chromosome5q21.2 and suggestive linkage at chromosomal regions 5q32-q33, 14q11.2, and 15q2.32 [24]. Despite efforts to screen numerous candidate genes in these loci, identification of causative genes (and mutations), however, remains elusive to a large extent. This may be due to the several limitations posed by linkage studies of complex diseases. The size of genetic effect, power of analysis to identify genes with small effects, presence of phenocopies with reduced penetrance, and various subclinical forms of keratoconus may be the reasons in many instances [45, 46]. With the advent of high-density SNP arrays and whole-genome/exome sequencing using the next-generation sequencing technologies it may now be possible to identify causal genetic variants in chromosomal regions exhibiting linkage in families of keratoconus.

## 5. Candidate Gene Analysis

Genetic risk factors for keratoconus have been difficult to identify because of the complex etiology of the disease. Among other approaches used to identify genetic components in families with suspected dominant forms of keratoconus, candidate gene analysis has also been employed to study keratoconus cohorts. Based on the underlying biological traits of the disease, candidate genes are predicted depending on their known biological functions and expression patterns relevant to the disease. Candidate gene approaches are particularly useful in studying complex multifactorial diseases and enables us to identify even small gene effects using large case-control cohorts. Similar approach has been commonly employed in keratoconus among different populations targeting potential genes involved in craniofacial and ocular development, extracellular matrix, collagens, apoptosis, and oxidative stress related pathways (Table 2).

Keratoconus exhibits overlapping pathophysiology with other corneal dystrophies [47]. Although clinically distinct, posterior polymorphous corneal dystrophy (PPCD) has been associated with keratoconus suggesting a common genetic pathway [48]. Mutations in* visual system homeobox 1* (*VSX1*; OMIM 605020) and* zinc-finger E-box binding homeobox 1* (*ZEB1*; OMIM 189909) genes have been previously implicated in PPCD [42, 49] and hence their role in keratoconus has been investigated.* VSX1* is a member of the paired-like homeodomain transcription factors family that plays a role in craniofacial and ocular development.* VSX1* is expressed by keratocytes only in injured corneas and is associated with fibroblastic transformation suggesting its role in response to wound and making it a potential candidate involved in corneal diseases [50]. The gene is mapped to chromosome 20p11.21. Since the first report by Héon and colleagues [49] numerous studies have evaluated the association of variants in* VSX1* and keratoconus [4, 51–56]. Two synonymous (rs56157240 and rs12480307) and one missense tag SNP (rs6050307) in the* VSX1* gene were found to be significantly associated with keratoconus in Han Chinese population [54]. Similarly, two heterozygous mutations (N151S and G160V) and an intragenic polymorphism (IVS1-11∗a allele) were significantly associated with increased risk of keratoconus in Korean patients [57]. The G160V accounted for 5.3% (13/249) of the study population. However, the results have been contradictory so far and this includes our own study in Saudi cohorts that failed to show any association [58–61]. Some of these common variants reported in* VSX1* are listed in Table 3. Although* VSX1* remains the most commonly studied gene the data from various populations suggests that variations in* VSX1* account for only 2-3% of keratoconus cases and may not have a major role in the molecular etiology of keratoconus reflecting the polygenic nature of this disease. To date there is only one recent study in unrelated European patients supporting the role of* ZEB1* mutations in keratoconus [34, 62] that needs to be replicated. Another gene associated with multiple types of corneal dystrophy includes an extracellular matrix gene,* transforming growth factor beta-induced* (*TGFBI*; OMIM 190180) [63]. A novel nonsense mutation (G535X) has been reported in a Chinese patient with keratoconus [64] but replication studies to confirm the role of* TGFBI* in keratoconus are lacking.

Based on disease pathogenesis of keratoconus, genetic alterations in collagen pathway that may affect corneal collagen structure/function have also been investigated without much success. No pathological mutations were reported in 104 European keratoconus patients in the* COL4A3* (OMIM 120070) and* COL4A4* (OMIM 120131) genes; however, significant allelic effects were seen for D326Y variant in* COL4A3* and M1237V and F1644F variants in* COL4A4* [65]. However, variants in* COL4A3* and* COL4A4* failed to show any association in Han Chinese population [54]. Similarly, variants in* COL4A1* (OMIM 120130) and* COL4A2* (OMIM 120090) genes failed to segregate with the disease in Ecuadorian families [66] and the role of* COL8A1* (OMIM 120251) and* COL8A2* (OMIM 120252) in keratoconus has also been excluded [67]. Keratoconus is associated with eye rubbing in atopic patients [68].* Filaggrin* (*FLG*; OMIM 135940) mutations are a strong genetic risk factor for atopic dermatitis. Mechanical injury to the keratoconic corneal epithelium results in keratocyte apoptosis via the interleukin 1 (IL1) pathway causing stromal thinning [69]. For these reasons,* FLG* and* IL1* have been suggested as candidate genes for keratoconus. Only five patients (5.6%) were found to be carriers of at least one* FLG *mutation of the two prevalent loss-of-function* FLG* alleles (R501X and 2282del4) indicating the need to look for other* FLG* mutations and/or other candidate genes [70].* IL1B* (OMIM 147720) promoter polymorphisms, rs1143627 (−31t>c) and rs16966 (−511c>t), were first reported to be associated with increased risk of keratoconus in Korean patients by Kim et al. [71]. The study also reported a decreased risk of the disease with an intronic* IL1A* (OMIM 147760) +376c>a polymorphism (rs2071376). Recently, Mikami and colleagues replicated the association of polymorphisms in* IL1B* (rs1143627, −31t>c and rs16944, −511c>t) and reported that the haplotype was associated with 1.72-fold increased risk of disease in Japanese keratoconus patients [72]. There are no functional studies yet to identify the role of these SNPs in the development of keratoconus. However, the data does suggest that polymorphisms in* IL1B* are important risk factors for susceptibility to keratoconus and must be investigated in other keratoconic populations.

In yet another recent study using linkage and candidate gene approach Li and colleagues identified a region of 5 Mb on chromosome 5q15 consisting of gene encoding* calpastatin* (*CAST*; OMIM 114090), an endogenous inhibitor of calpains [73]. SNP (rs4434401) located in* CAST* gene was associated with both familial and sporadic keratoconus making it an interesting candidate to be tested in other ethnic groups. Other candidate genes screened in keratoconus cohorts include* TIMP3* (OMIM 188826),* SPARC* (OMIM 182120), and* SOD1* (OMIM 147450). The tissue inhibitors of metalloproteinases (TIMPs) are natural inhibitors of matrix metalloproteinases and the balance between the two regulates remodeling of the extracellular matrix.* TIMP3* has been shown to be differentially expressed in keratoconic corneas [74]. However, no pathogenic variants have been identified in keratoconus patients [51].* SPARC* is localized to a chromosomal region 5q31.3-g32 that was suggestive of linkage in familial keratoconus [41] making it an interesting candidate. Three novel missense mutations (E63K, c.187g>a; M92I, c.276g>a; and D219E, c.657c>a) have been reported in Italian patients with keratoconus that were not detected in 200 controls; however, it is not clear yet whether these are rare polymorphisms or causative mutations and need further investigation [51]. Since oxidative stress has been hypothesized to play a role in the etiology of keratoconus [75, 76] and given the association of trisomy 21 (Down syndrome) with keratoconus, association of variants in* SOD1 *gene localized on chromosome 21 has also been investigated. Although an intronic deletion in* SOD1* was found to segregate in two small families with keratoconus [77], this finding has not been consistent in additional cohorts, including in Saudi cohorts [78], and so far accounts for less than 1% of keratoconus cases.

## 6. Keratoconus and the Mitochondrial Connection

It was previously reported that mitochondrial oxidative stress in Tet-mev-1 mice causes excessive apoptosis in several tissues leading to precocious age-dependent corneal physiological changes, delayed corneal epithelialization, decreased corneal endothelial cells, thickened Descemet's membrane, and thinning of parenchyma with corneal pathological dysfunctions such as keratitis, Fuchs' corneal dystrophy (FCD), and probably keratoconus [79]. Under TEM, swelling of the mitochondria was observed in keratoconus corneal tissues [80]. Keratoconus corneas exhibited more mitochondrial DNA (mtDNA) damage than normal corneas [75]. Keratoconus fibroblasts had increased basal generation of reactive oxygen species and were more susceptible to stressful challenges (low-pH and/or H_2_O_2_ conditions) than were normal fibroblasts [81]. Additionally, cultured keratoconus fibroblasts have an inherent, hypersensitive response to oxidative stress that involves mitochondrial dysfunction and mtDNA damage [81]. As a result, it was suggested that keratoconus fibroblast hypersensitivity may play a role in the development and progression of keratoconus [82]. A large number of variations including two novel frameshift mutations in mitochondrial complex I (*ND1-6*) gene of the mitochondrial genome have been reported in keratoconus patients negative for* VSX1* mutations [83]. Similarly, Abu-Amero and colleagues had recently shown that mtDNA mutations were present in keratoconus patients from Saudi Arabia [84]. In addition, our recent study showed that the mean relative mtDNA content was found to be significantly higher in patients with keratoconus than the normal control subjects [85]. Future studies in large cohorts of multiple ethnic populations are needed to establish the role of oxidative stress in keratoconus and the relevance of mitochondrial genes which in parts may provide insights into the pathophysiological mechanisms of this complex disease.

## 7. Keratoconus Associated with Other Disorders

Keratoconus has been associated with numerous systemic and ocular disorders, including Leber congenital amaurosis [86, 87], X-linked hypohidrotic ectodermal dysplasia with mutation in* EDA* (OMIM 300451) gene [88], Williams-Beuren syndrome [89], brittle cornea syndrome [90], Costello syndrome [91], intellectual disability [92], and other corneal dystrophies (PPCD and Fuchs' endothelial dystrophy) [62, 93]. Individuals with Down syndrome have 10–300-fold higher prevalence of keratoconus [1, 94]. Other chromosomal abnormalities include Turner syndrome [95], chromosome 13 ring anomaly [96], and translocation 7;11 [97]. Similarly, individuals with connective tissue disorders such as Marfan syndrome [98], Ehlers-Danlos syndrome [99], and mitral valve prolapse [100] also show an increased prevalence of keratoconus. Such observations imply that either these disorders may provide environmental triggers for manifestation of keratoconus or they share a common underlying genetic mechanism(s). However, the caveat to this plausibility is that the mutations in the causative genes for such disorders have not been found to be enriched or causative in isolated keratoconus cases. Therefore, the exact relevance of increased prevalence of keratoconus associated with these genetic disorders is still not known.

## 8. Genome-Wide Association Studies

Genome-wide association studies (GWAS) in case-control cohorts provide a powerful platform to identify common risk variants in complex genetic disease. These studies identify SNP(s) that are in linkage disequilibrium with causative variants and require large population-based samples to achieve a genome-wide significance (*P* < 5 × 10^−8^). To this end, GWAS has been performed in keratoconus, albeit in relatively small number of patients. A recent segregation analysis in unrelated sporadic keratoconus families indicated that keratoconus is a complex non-Mendelian disease with low genotype-phenotype correlation and suggested the use of GWAS, epigenetics, and pathway analyses in nonfamilial keratoconus [101]. Using GWAS, Burdon and colleagues identified a SNP (rs3735520) at the* HGF* (OMIM 142409) locus to be associated with keratoconus in cohorts from Australia, USA, and Northern Ireland [102]. In another GWAS, Li and colleagues identified a SNP (rs4954218) located near* RAB3GAP1* (OMIM 602536) as a potential susceptibility locus for keratoconus in Caucasian cohorts from USA [103]. In both these GWAS the results did not achieve a genome-wide significant *P* value. The findings of SNP rs4954218 have been replicated in an Australian Caucasian cohort and represent a strong candidate for keratoconus [104].

Li and colleagues performed a two-stage genome-wide linkage scan in keratoconus families and identified a locus at chromosome 5q23.2, overlapping the gene encoding* lysyl oxidase* (*LOX*; OMIM 153455) [41].* LOX* is involved in corneal collagen and elastin cross-linking [105]. Artificial collagen fibre cross-linking following riboflavin and UV light exposure is a procedure currently being tested for the treatment of keratoconus and therefore this gene may have therapeutic implications [106]. Two SNPs (rs10519694 and rs2956540) in* LOX* showing nominal genome-wide significance were associated with keratoconus by family-based association testing that were also found to be significantly associated with keratoconus in case-control cohorts [107]. Other studies so far have provided conflicting evidence for the role of* LOX* in keratoconus [51, 108].

An alternative approach to case-control GWAS would be to test for association with intermediate phenotypes and identify the heritable quantitative trait loci. An elegant example of this approach was demonstrated by Lu and colleagues to identify multiple loci associated with central cornea thickness (CCT) and keratoconus [109]. In a large (>20,000) European and Asian population-based sample the study reported 16 new loci associated with CCT at genome-wide significance. The authors then investigated the association of these CCT-linked loci with risk of keratoconus in 874 cases and 6,085 controls. This meta-analysis identified six SNPs within or nearby* FOXO1 *(rs2721051),* FNDC3B *(rs4894535),* RXRA-COL5A1* (rs1536482),* MPDZ-NFIB* (rs1324183),* COL5A1* (rs7044529), and* BANP*-*ZNF469* (rs9938149) genes/loci that were strongly associated with the risk of keratoconus. Interestingly, a deleterious mutation in* ZNF469 *(OMIM 612078) is known to cause brittle cornea syndrome which is associated with extremely thin and fragile cornea [110]. However, the GWAS showed an unexpected effect direction for SNP rs9938149 in* BANP*-*ZNF469*, with the CCT-increasing allele leading to increased risk for keratoconus, implying that, in addition to alternative mechanisms which may be specific for keratoconus, part of the genetic predisposition to keratoconus is mediated through the genes underlying CCT [109]. The association of SNPs rs1324183 and rs9938149 with keratoconus has been replicated in an independent Australian cohort; however, their association was via a noncorneal curvature route [111]. Using a similar approach Li and colleagues identified polymorphisms (rs1536482 and rs7044529) in the* COL5A1* (OMIM 120215) region which regulate normal variation in CCT and may play a role in corneal thinning associated with keratoconus [112].

## 9. Chromosomal Copy Number Alterations

Copy number variation (CNV) is also a significant molecular mechanism in Mendelian diseases. These variants can be detected on a genome-wide level using microarray-based comparative genomic hybridization. In our previous study in 20 Saudi patients with isolated nonfamilial keratoconus we did not detect any chromosomal variations that would account for the disease [113].

## 10. Gene Expression Studies

In addition to the numerous studies focused on identifying genes using DNA-based approaches, few studies have also investigated the mRNA transcriptome to identify functional genes and the disease pathways. Microarray analysis of 11 keratoconus and 8 normal corneal epithelium samples resulted in identification of 47 genes that were upregulated and 9 genes that were downregulated. The* keratin 72 *(*KRT72 *OMIM 608246) gene was the most upregulated (5.2-fold) gene identified in this study [114]. Another study identified genes in apoptosis pathway that were differentially expressed suggesting a role in stromal thinning [115]. Mootha and colleagues reported a 212-fold decrease in* alcohol dehydrogenase 1B (class I) beta polypeptide *(*ADH1B;* OMIM 103720) mRNA levels in cultured keratocytes [116]. Similarly, to identify differentially expressed genes in human keratocytes, Lee et al. performed PCR-based differential analysis on cultured corneal stromal fibroblasts from normal and keratoconic corneas. The study reported upregulation of* bone morphogenetic protein 4* (*BMP4*; OMIM 112262),* cofilin 1* (*CFL1*; OMIM 601442), and* JAW1-related protein* (*MRVI1*; 604673) and downregulation of* actin, alpha 2* (*ACTA2*; OMIM 102620),* gene rich cluster*,* C 10 gene* (*GRCC10*; OMIM 615140),* tissue inhibitor of metalloproteinases 1* and* 3* (*TIMP1*; OMIM 305370 and* TIMP3*), and* somatostatin receptor 1* (*SSTR1*; OMIM 182451) [74]. A novel cornea-expressed gene,* aquaporin 5* (*AQP5*; OMIM 600442), was found to be downregulated in keratoconic corneas as compared to controls [117]. In another study using pooled patients corneas, microarray analysis identified 87 differentially expressed genes that belonged to pathways of apoptosis and regulation of cellular differentiation and proliferation. Of these, a majority of 69 genes were found to be downregulated, controlled by transcription factor AP1 [118].

Overall, the data from gene expression studies suggest a role of genes involved in apoptosis, cellular differentiation, and proliferation pathways which supports the proteomic data. Unfortunately, none of the studies help validate genes identified from DNA-based genetic studies with the exception of extracellular matrix genes (*TIMP1* and* TIMP3*). In addition, the identification of* AQP5* could not be replicated in another study [119]. However, the role of other genes (e.g.,* BMP4*,* CFL1*) identified by differential expression analysis as a potential mediator of keratoconus requires further studies. Gene expression is known to be tissue and cell specific. The use of different tissue and cell types, forme fruste of keratoconus, and methods used for expression studies may be the likely cause(s).

## 11. Conclusion

There is sufficient data to suggest that keratoconus has a major genetic predisposition. Current understanding supports a complex etiology involving both genetic and environmental factors. A number of genetic susceptibility loci have been implicated in keratoconus and there exists genetic heterogeneity rather than a single major gene-effect responsible for development and progression of keratoconus. Family-based studies have led to the identification of* MIR184* and* DOCK9* genes in cases with familial keratoconus; however, the replication of locus identified in a specific keratoconus family in other families (even in homogeneous population) has been very limited. Identification of a mutation in* MIR184* in keratoconus opens a new area of research to study miRNA regulation in eye diseases. Despite numerous efforts potentially pathogenic variants have been identified in only small number of keratoconus patients and a majority of causative genes and mutations remain to be unraveled. Although* VSX1* only accounts for rare cases, it still remains the most studied keratoconus gene. However, it is unclear whether and how mutations in* VSX1* contribute to the pathogenesis of keratoconus. Other candidate genes including* SOD1*,* ZEB1*,* TGFB1*,* FLG*, interleukin, and collagen may have a role to play in the pathogenesis of keratoconus and need further elucidations. Similarly, other candidate genes identified by GWAS including* HGF*,* RAB3GAP1*,* LOX*,* MPDZ, NFIB*,* BANP*, and* ZNF469* may be important risk factors for keratoconus and require replication studies in other populations. Gene expression analyses are a valuable tool in addition to other approaches in identifying candidate genes and to elucidate the pathogenesis of keratoconus. In addition, identification of heritable quantitative trait loci can provide an alternative approach for selection and analysis of candidate genes in keratoconus [109] and has been successfully demonstrated in Australian cohort [111]. The subclinical forms of keratoconus may be a confounding factor and complicate the identification of common genetic defects. It is therefore important to include clinically well-defined and accurately classified patients to minimize phenotypic differences. With increased utilization of high-density SNP arrays and whole-genome/exome sequencing technologies it will now be possible to apply genome-wide approach to identify causal genetic variants in both familial and sporadic forms of keratoconus and at a more rapid pace; until then the molecular pathogenesis of keratoconus remains heterogeneous.

## Figures and Tables

**Table 1:** Loci identified in keratoconus patients by linkage analysis and identity-by-descent approach.

Locus	LOD score	Candidate genes excluded^‡^	Population	Reference
1p36, 8q13-q21	3.4	*ENO1, CTNNBIP1, PLOD1, UBIAD1, SPSB1, *and *TCEB1 *	Australian	[18]
2p24	5.13	—	Caucasian and Arab	[40]
3p14-q13	3.09	*COL8A1 *	Italian	[43]
4q, 5q, 12p, and 14q (suggestive)	—	—	Caucasian and Hispanic	[41]
5q14.1-q21.3	3.53	—	Americans	[39]
5q21, 5q32-q33, and 14q11 (suggestive)	—	—	Italian	[24]
9p34	4.5	—	Caucasian and Hispanic	[41]
14q24.3	3.58	*VSX2 *	Mixed	[42]
16q22-q23	4.1	—	Finnish	[23]
20q12^†^	—	*MMP9 *	Australian (UK descent)	[38]

^†^Mapped using identity-by-descent approach.

^‡^No mutations were found in these screened candidate genes.

**Table 2:** Some of the genes reported in keratoconus using different genomic approaches.

Genes	Method	Population	Reference
*MIR184 *	Linkage	Northern Irish	[30]
*DOCK9 *	Linkage	Ecuadorian	[36]
*VSX1 *	Candidate gene	European	[49]
*ZEB1 *	Candidate gene	European	[62]
*TGFB1 *	Candidate gene	Chinese	[64]
*COL4A3/COL4A4 *	Candidate gene	European	[65]
*FLG *	Candidate gene	European	[70]
*IL1A *	Candidate gene	Korean, Chinese	[54, 71]
*IL1B *	Candidate gene	Korean, Japanese	[71, 72]
*CAST *	Linkage/candidate gene	Americans	[73]
*SOD1 *	Candidate gene	Americans	[77]
*HGF *	GWAS	Australian, Americans	[102]
*RAB3GAP1 *	GWAS	Americans, Australian	[103, 104]
*LOX *	Linkage/GWAS	Americans	[107]
*MPDZ-NFIB, BANP-ZNF469 *	GWAS/candidate gene	European/Asian and Australian	[109, 111]
*COL5A1 *	Linkage/GWAS	Americans	[112]
*KRT72 *	Gene expression	European	[114]
*TIMP1, TIMP3, BMP4,*and* CFL1 *	Gene expression	Korean	[74]

**Table 3:** Common *VSX1* variants reported in patients with keratoconus.

SNP ID	Amino acid change	Clinical significance	Population	Reference
rs74315436	Leu17Pro	Pathogenic	European	[51, 55]
rs6050307	Arg131Ser	Pathogenic	Han Chinese	[54]
rs140122268	Asp144Glu	Unknown	European	[56]
rs74315434	Leu150Met	Unknown	Canadian, Americans, and European	[49, 51, 60]
rs74315433	Gly160Asp	Pathogenic	European	[55, 56]
—	Gly160Val	Pathogenic	Korean	[57]
rs74315432	Arg166Trp	Pathogenic	Canadian, Iranian	[49, 52]
rs148957473	His244Arg	Unknown	Canadian, Americans, and Iranian	[49, 52, 60]
VAR_014248	Pro247Arg	Unknown	Canadian, European	[49, 55]
